# Piperine as a modulator of cancer hallmarks: mechanistic insights and therapeutic potential

**DOI:** 10.3389/fonc.2026.1810344

**Published:** 2026-04-07

**Authors:** Wamidh H. Talib, Mohanad Jawad Kadhim, Hadeel Shaher Al Junaidi, Assia BenBraiek, Ward M. Mohanna, Yara A. Alfaleet, Rawan W. Hadi, Douglas Law, Akila Prashant, Krisztina Takács, Imre Soós

**Affiliations:** 1 Faculty of Allied Medical Sciences, Applied Science Private University, Amman, Jordan; 2 Department of Applied Biotechnology, College of Biotechnology, Al-Qasim Green University, Babylon, Iraq; 3 Medical and Clinical Laboratory Technology, Faculty of Allied Medical Sciences, Applied Science Private University, Amman, Jordan; 4 Faculty of Pharmacy, Al-Azhar University of Gaza, Gaza, Palestine; 5 Department of Clinical Nutrition and Dietetics, Applied Science Private University, Amman, Jordan; 6 Faculty of Health and Life Sciences, Inti International University, Nilai, Malaysia; 7 Department of Biochemistry, JSS Medical College, JSS Academy of Higher Education & Research, Mysuru, Karnataka, India; 8 Department of Basics of Health Sciences, Institute of Basics of Health Sciences, Midwifery and Health Visiting, Faculty of Health Sciences, University of Pécs, Pécs, Hungary; 9 Department of Sport Science, Institute of Physiotherapy and Sport Science, Faculty of Health Sciences, University of Pécs, Pécs, Hungary

**Keywords:** angiogenesis, anticancer, cancer, cancer hallmarks, metastasis, piperine

## Abstract

**Background:**

Cancer processing relies on a range of biological capabilities referred to as cancer hallmarks. There is growing interest in natural bioactive compounds that have the potential to modulate these hallmarks. Piperine, an alkaloid isolated from *Piper nigrum* and *Piper longum*, has shown various biological effects, including anticancer activity in preclinical studies.

**Methods:**

The present review summarizes *in vitro* and *in vivo* studies regarding the impact of piperine on various cancer attributes. The available Literature was thoroughly analyzed to identify evidence for the molecular basis by which piperine modulates cell growth, apoptosis, angiogenesis, invasion and metastasis, inflammation, and oxidative stress in various types of cancer.

**Discussion:**

The compound has been shown to inhibit the proliferation of various cancer cell lines and may enhance the effectiveness of conventional chemotherapy agents by improving drug bioavailability and reducing resistance. Moreover, piperine may target several hallmarks of cancer that influence cancer progression and metastasis, including angiogenesis, sustained proliferation, genomic instability, resistance to cell death, immune evasion, and disrupted redox homeostasis. These multifaceted effects of piperine are believed to be mediated through the modulation of signaling pathways such as NF-κB, PI3K/Akt, MAPK, and STAT3. Although these results are encouraging, the therapeutic use of piperine is limited by its poor solubility, low bioavailability, and lack of clinical research.

**Conclusion:**

Piperine is a promising multitarget compound that can influence important cancer characteristics in preclinical studies. Nevertheless, additional research is needed to overcome formulation issues, assess toxicity, and conduct well-structured clinical trials to confirm its potential as an effective anticancer treatment.

## 1 Introduction

Cancer is a fatal disease that affects human health globally and causes considerable economic and human losses worldwide (1). It can develop in any organ and is mainly caused by genetic mutations that disrupt the normal regulation of the cell cycle. These mutations allow cells to grow uncontrollably, avoid programmed cell death (apoptosis), and invade nearby tissues. Without treatment, malignant cells can spread to distant organs through metastasis, impairing overall bodily functions (2). Cancer remains the second leading cause of death worldwide. In 2022, about 20 million new cancer cases were diagnosed, and 9.7 million people died from the disease globally. By 2050, the number of cancer cases is expected to rise to 35 million, based solely on projected population growth (3). Despite advances in detection and treatment methods, cancer continues to be one of the most well-known and challenging diseases to cure in humans. Various treatment strategies, including radiotherapy, chemotherapy, surgery, and targeted therapies, have improved tumor and metastasis management; however, many adverse effects have been observed, such as multidrug resistance to chemotherapy and toxicity to healthy tissues (4). Additionally, side effects can limit the use of anticancer drugs and negatively impact patients’ quality of life (5). Although significant progress has been made in understanding cancer through advanced research and sophisticated diagnostic and therapeutic technologies, discovering new therapeutic agents remains a vital and active area of cancer research. In light of evolving cancer treatment options, there has been growing interest in natural compounds derived from plants due to their potential as anticancer agents (6), along with their low toxicity, chemical diversity, and ability to target various cancers (7). Many medicinal plants and isolated phytochemicals have gained considerable attention because they can target diverse cancer cell populations, influence key signaling pathways involved in cancer progression at different stages, and generally have a favorable safety profile (8, 9).

Piperine (C_17_H_19_NO_3_) is a secondary plant alkaloid. This solid, weakly basic compound is insoluble in water and initially tasteless but produces a scorching sensation afterward. It is classified as a vanilloid molecule, similar to capsaicin found in chili peppers, which imparts a spicy sensation. Plants containing piperine have been traditionally used in medicine and culinary practices worldwide (10, 11). With 1,002 publications in the past decade, piperine has gained significant attention for its health benefits (12–16). Since its discovery in 1820, numerous biological activities have been reported. Various studies have demonstrated its antibacterial, anti-inflammatory, anticancer, analgesic, anticonvulsant, antiulcer, gastroprotective, antioxidant, insecticidal, and antiseptic effects (17–19). Piperine modulates the metabolism and transport of bioactive compounds to enhance their absorption and bioavailability (20). It acts as an inhibitor of the primary hepatic drug-metabolizing enzyme (CYP3A4) and the drug efflux pump (P-glycoprotein). This dual action increases the plasma concentration of chemotherapeutic drugs, thereby enhancing their anticancer efficacy. However, this also elevates systemic toxicity, leading to severe side effects such as neutropenia and peripheral neuropathy (21, 22).

This review aims to compile and analyze existing research to elucidate the role of piperine in cancer treatment, focusing on its direct and indirect effects on the hallmarks of cancer. Additionally, the review will examine previous studies on piperine’s pharmaceutical formulations and the challenges associated with its clinical use.

Although growing evidence supports the antitumor effects of piperine, certain limitations remain. Most findings are derived from *in vitro* and *in vivo* preclinical studies, with limited data on its pharmacokinetics, optimal dosage, and long-term safety in humans. Additionally, variations in experimental designs and cancer models complicate direct comparisons across studies. Importantly, the lack of clinical research represents a significant gap in translating these findings into clinical practice, which must be addressed for piperine to progress toward clinical application.

## 2 literature search strategy

To identify relevant studies on the biological activities, pharmacological properties, and anticancer potential of piperine, a systematic literature search was conducted. Major scientific databases, including PubMed, Scopus, and Web of Science, were utilized for this search.

The search strategy utilized combinations of the following terms: “piperine,” “*Piper nigrum*,” “anticancer activity,” “chemotherapy sensitization,” “multidrug resistance,” “apoptosis,” “phytochemicals in cancer therapy,” “piperine pharmacology,” and “piperine molecular mechanisms.” Boolean operators (AND/OR) were applied to refine the search and identify relevant articles. Only studies published in English between 2000 and 2025 were included. Both original research articles and review papers examining the biological, pharmacological, and medicinal properties of piperine were considered. Additionally, the reference lists of selected articles were manually screened to identify further relevant studies.

Studies reporting experimental, clinical, or mechanistic evidence regarding piperine were included, particularly those focusing on its pharmacological properties, anticancer activity, or therapeutic applications. Excluded were studies unrelated to piperine, articles lacking sufficient scientific data, conference abstracts without full texts, and non–peer-reviewed sources. The initial search yielded over 300 publications, which were then filtered for relevance based on titles and abstracts. Approximately 133 studies were selected and incorporated into this review after removing duplicates and irrelevant articles.

## 3 Piperine: source, chemistry, and pharmacological profile

### 3.1 Chemical structure and formation

**Figure 1:**
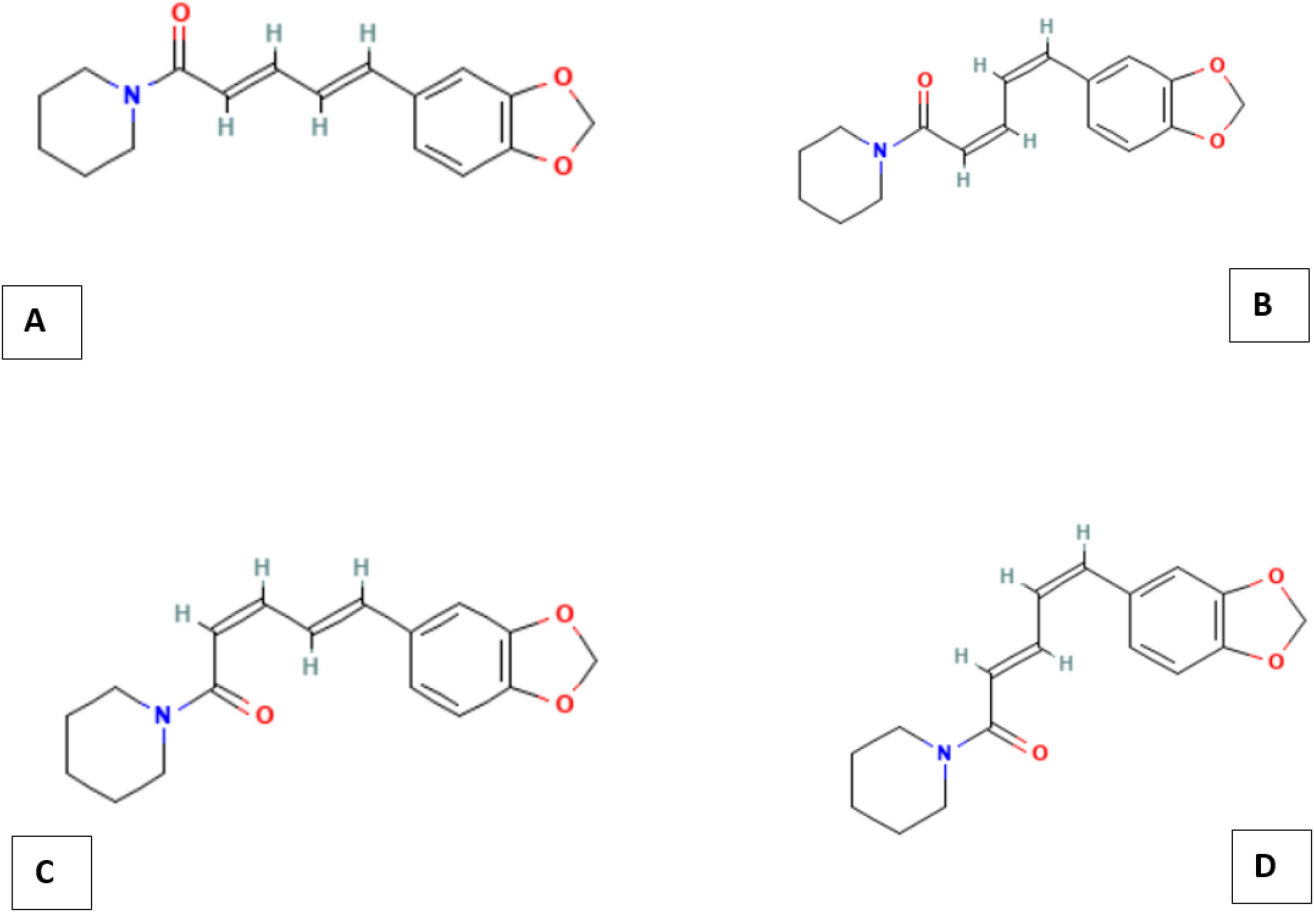
Chemical Structures of the four isomers of piperine: **(A)** piperine, **(B)** chavicine, **(C)** isopiperine, and **(D)** isochavicine. Structural data were retrieved from the PubChem database (NCBI) (27).

Black pepper contains a significant bioactive compound, including piperine, which is the most abundant pungent principle present in black pepper (23). Originally extracted in the early 1800s, the chemical structure of piperine was progressively elucidated between 1882 and 1894 (24). Its IUPAC name is (2E,4E)-5-(benzo[d][1,3]dioxol-5-yl)-1-(piperidin-1-yl)penta-2,4-dien-1-one. The pungency of black pepper is primarily due to piperine, an amide that activates the TRPV-1 receptor, similar to capsaicin in chili peppers. The piperine molecule contains a dioxymethylene ring, an aliphatic olefin chain, and an amide group (25) (**Figure 1**). Its organic synthesis is well studied; however, the biosynthetic pathway in black pepper remained unclear until recent discoveries. Early studies linked piperine’s piperidine ring to L-lysine and cadaverine, while its aromatic structure has been suggested to derive from the phenylpropanoid pathway. More recent research has identified key enzymes involved in piperine biosynthesis, including piperoyl-CoA ligases and a cytochrome P450 oxidoreductase (CYP719A37). Amide formation is believed to be the final step, potentially catalyzed by a BAHD-type acyltransferase, similar to capsaicin synthase (26).

### 3.2 Sources of piperine

Piperine is a naturally occurring alkaloid extracted from several species within the Piperaceae family, including *Piper nigrum* (black pepper), *Piper longum* (long pepper), *Piper chaba*, and *Piper guineense* (28). The piperine content in black pepper is estimated to range from 2% to 10% (24). Black pepper contains four isomeric forms of piperine: the trans-trans isomer (1, piperine), the cis-trans isomer (2, isopiperine), the cis-cis isomer (chavicine, 3), and the trans-cis isomer (isochavicine, 4). The arrangement of the double bonds in the side chain of these isomers varies, which could affect their stability and biological properties (**Figure 1**) (23).

### 3.3 Method of extraction

Piperine is a pungent alkaloid and a secondary metabolite extracted from plants of the *Piper* genus. Various extraction methods are available and are selected based on the scale and purpose of the extraction because it offers effective extraction in mild conditions, low solvent contamination, and high selectivity (11). Traditional techniques include Soxhlet or modified Soxhlet extraction, ethanol extraction, and maceration using glacial acetic acid. In order to improve extraction efficiency and shorten extraction times, advanced extraction methods have recently been developed, including ionic liquid-based ultrasonication-assisted extraction, microwave-assisted extraction, microwave reflux extraction, ultrasound-assisted extraction (UAE), supercritical fluid extraction (SFE), and extraction using the Naviglio extractor. The solvent system and extraction conditions have an impact on reported yields. For instance, it has been reported that ethanol extraction yields about 3.2% piperine, microwave reflux extraction yields between 2.05 and 4.27%, and ultrasound-assisted extraction yields about 5.8 mg/g. Supercritical fluid extraction (SFE), one of the more advanced methods, has shown greater efficiency; under optimal conditions, reported yields have reached up to 7.6% (w/w) (23).

Emerging extraction techniques are being explored to obtain higher-purity piperine, with Supercritical Fluid Extraction (SFE) widely recognized as one of the most efficient and commonly adopted methods. High-performance liquid chromatography (HPLC) and high-performance thin-layer chromatography (HPTLC) enable precise detection of piperine at very low concentrations, ranging from nanogram to picogram levels, facilitating its medicinal applications and pharmacokinetic studies (11). In addition, a recent comparative study evaluated different extraction techniques for piperine: Soxhlet extraction yielded the highest amount, ultrasonic extraction produced the highest phenolic content, and the reflux method demonstrated the best antioxidant activity (29). These results show that the extraction method, solvent polarity, and operational parameters have a significant impact on the phytochemical composition and extraction efficiency.

### 3.4 Chemical and physical properties

Piperine is an alkaloid with the chemical formula C17H19NO3 and a molecular weight of 285.34 Daltons. It belongs to the largest class of plant secondary metabolites, known as alkaloids (11).

Due to its lipophilic nature, this yellow solid compound is soluble in ether and other oils but insoluble or slightly soluble in water. It exhibits weak basicity and is initially tasteless, gradually developing a burning sensation. Piperine has a melting point of approximately 129 °C and a boiling point ranging from 498 to 499 °C (28).

### 3.5 Piperine toxicity

Piperine is primarily consumed as a spice and nutrient enhancer. Based on studies, the No Observed Adverse Effect Level (NOAEL) is established at 5 mg/kg/day in rats, indicating that doses up to this level do not produce any adverse effects. This suggests that dietary consumption is generally safe; however, excessive intake may cause irritation (28).

Toxicity studies in animals indicate that piperine is non-toxic at subacute doses up to 100 mg/kg body weight in rodent animals. However, lethal dose (LD_50_) values for intravenous and oral administration suggest potential toxicity at higher doses. Some studies associate piperine with liver damage, while others emphasize its protective effects (28).

Genotoxicity studies present conflicting results between *in vitro* assays and *in vivo* rodent studies; however, most findings support the safety of piperine. Furthermore, studies on reproductive toxicity in Swiss albino mice showed that although lower doses seem safe, higher doses may affect testicular function and decrease sperm production (30).

Immunotoxicity studies in Swiss albino mice have shown that consecutive administration of piperine at higher doses (2.25 and 4.5 mg/kg) for five days results in a reduction in total white blood cell count, an increase in neutrophils, and suppression of B-lymphocyte responses to stimuli. Additionally, these higher doses cause a decrease in the weight of the spleen, thymus, and lymph nodes. However, at a lower dose (1.12 mg/kg), piperine appears to be immunologically safe (11).

### 3.6 Pharmacological activity

**Figure 2:**
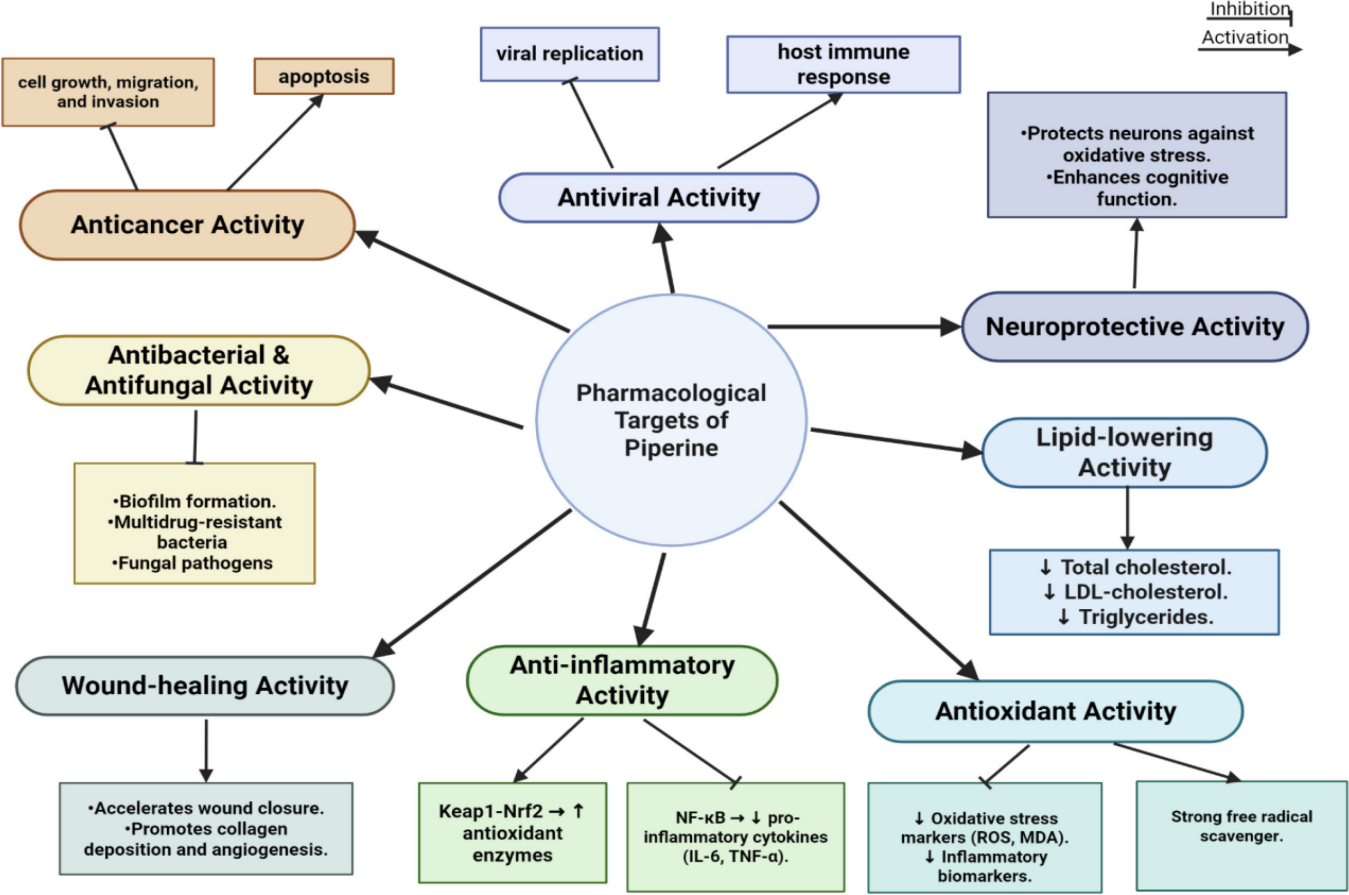
Schematic representation of the major pharmacological targets of piperine. The figure was created using BioRender.com.

Piperine exhibits a wide range of pharmacological activities, making it a highly promising candidate for drug development. It demonstrates potent anticancer effects by inhibiting cell growth, migration, and invasion, with structural modifications further enhancing its cytotoxic potential (31). Additionally, piperine exhibits antiviral properties, demonstrating strong efficacy against influenza A virus subtype H1N1, SARS-CoV-2, and the largemouth bass virus (LMBV) (32). Its anti-inflammatory activity is evident in conditions such as acute pancreatitis, psoriasis, UV-induced skin damage, and sciatica, where it modulates key inflammatory pathways including NF-κB, MAPK, and Keap1-Nrf2 (24, 33). Moreover, piperine-based compounds exhibit insecticidal activity, effectively targeting pests such as *Aedes aegypti* and *Ostrinia furnacalis* (Asian corn borer) by disrupting chitin metabolism (32, 34). Piperine also exhibits antibacterial and antifungal properties, inhibiting biofilm formation and multidrug-resistant bacterial strains (e.g., *Pseudomonas aeruginosa* and *Vibrio cholerae*), as well as fungal pathogens such as *Candida* and *Trichophyton* species (35, 36). Beyond these effects, piperine shows neuroprotective activity and promotes wound healing (28, 37). Furthermore, in experimental models of obesity and dyslipidemia, piperine has been demonstrated to lower circulating levels of total cholesterol, low-density lipoprotein cholesterol, and triglycerides. It also has positive effects on lipid metabolism (38, 39). Clinical studies assessing curcumin-piperine supplementation have shown decreases in circulating triglycerides and total cholesterol in people with metabolic disorders, and piperine has been reported to improve lipid metabolism (40, 41). Piperine also exhibits strong antioxidant properties, contributing to the reduction of oxidative stress and inflammatory markers (42). Collectively, these pharmacological properties underscore piperine’s significant potential in therapeutic applications and drug discovery (**Figure 2**).

### 3.7 Metabolism and bioavailability

**Figure 3:**
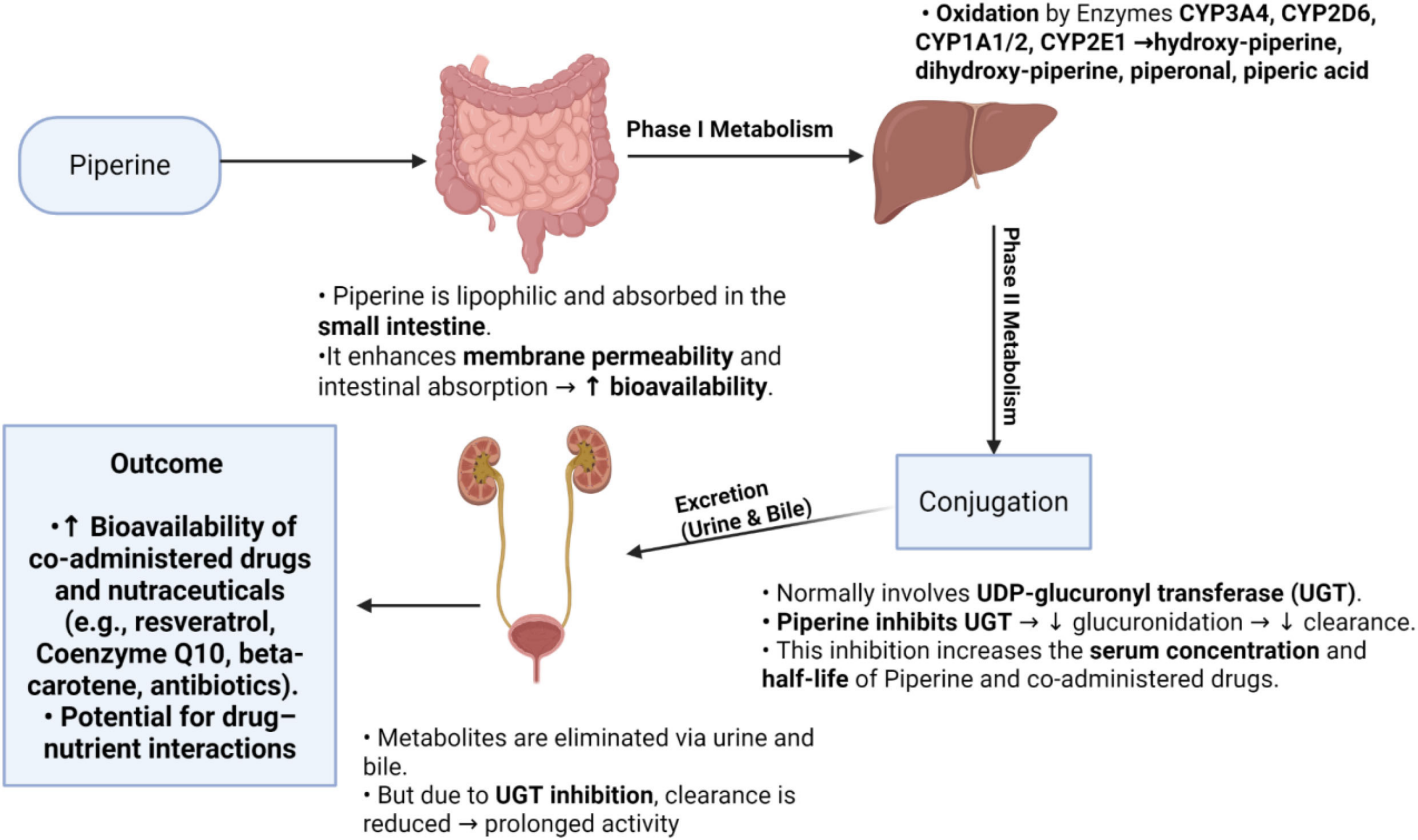
Diagram illustrating the metabolism of piperine in the human body. Piperine is primarily metabolized by cytochrome P450 enzymes in the liver, resulting in various metabolites that influence its pharmacokinetics, biological activity, and bioavailability. The figure was created using BioRender.com.

Piperine plays a crucial role in medication metabolism by modulating enzyme activity and increasing bioavailability. It inactivates CYP3A enzymes and enhances the anti-inflammatory properties of resveratrol. Additionally, piperine improves membrane permeability and absorption site dynamics, thereby increasing the serum half-life and efficacy of coenzyme Q10 and beta-carotene (**Figure 3**). However, it also inhibits UDP-glucuronyl transferase, CYP3A4, and aryl hydrocarbon hydroxylase, which reduces drug metabolism and consequently increases the bioavailability of antibiotics, anti-inflammatory drugs, and phytochemicals. Due to these properties, nanoparticles may further enhance piperine absorption and its therapeutic benefits (28, 43).

Despite its potential benefits, its lipophilic nature limits its bioavailability, necessitating further research into consumption patterns and the development of improved delivery systems for pharmaceutical applications.

## 4 Role of piperine in targeting cancer hallmarks

### 4.1 Role of piperine in genomic instability

Genomic instability is a fundamental hallmark of all cancer cells, enabling malignant cells to accumulate mutations and evade regulatory mechanisms. It can be induced by various mechanisms, including telomerase dysfunction, centrosome amplification, epigenetic modifications, and DNA damage (44).

**Figure 4:**
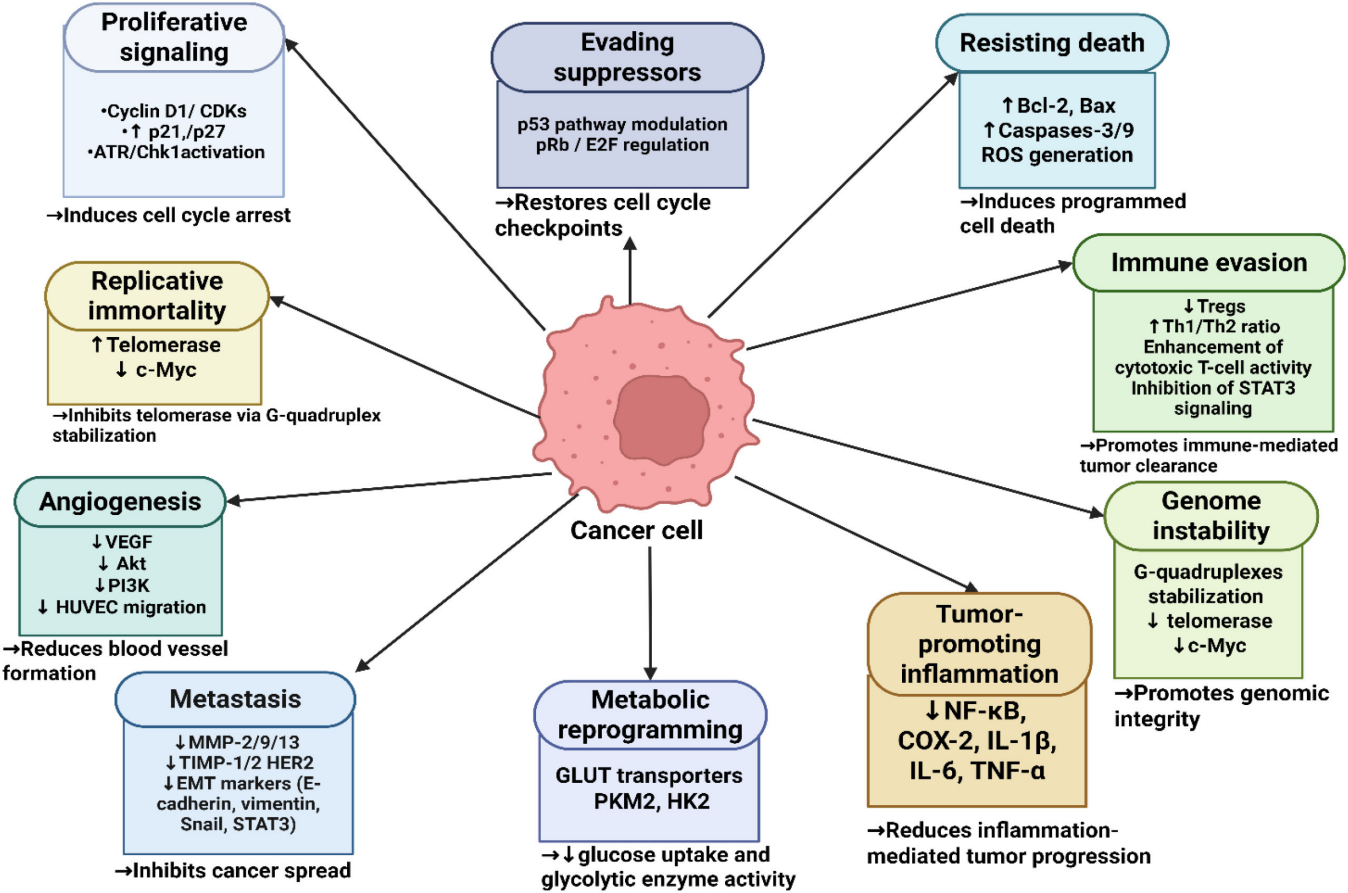
Schematic representation of the molecular mechanisms by which piperine targets major cancer hallmarks and signaling pathways to inhibit tumor growth and progression. The figure was created using BioRender.com.

Oncogenes and tumor suppressor genes regulate specific checkpoints that maintain genome integrity during the normal cell cycle. However, cancer cells can alter the function of these checkpoints, leading to tumorigenesis and the promotion of uncontrolled cell growth (45). Major hallmarks of cancer are summarized in **Figure 4**.

G-quadruplexes are non-canonical DNA structures formed by guanine-rich sequences. They are particularly prevalent in telomeres and regulatory regions of oncogenes such as c-kit and c-myc, making them promising targets for anticancer drugs (46). Recent studies have revealed that piperine can inhibit cancer development by targeting human G-quadruplex DNA sequences. Telomeric G-quadruplexes play a crucial role in inhibiting telomerase, an enzyme that is overactive in most cancers and essential for maintaining telomere length and cancer cell immortality. Piperine reduces telomerase activity by promoting the formation of G-quadruplexes, highlighting its potential as an anticancer agent (46, 47). Additionally, piperine targets the c-myc promoter G-quadruplex. Overexpression of c-myc, which regulates cell growth and proliferation, can drive tumor progression. A guanine-rich region (Pu27) in the c-myc promoter (NHE III1) forms a G-quadruplex that silences c-myc transcription. In cancer cells, small-molecule ligands such as piperine can downregulate c-myc expression by stabilizing this structure. Piperine, a strong ligand, binds to and stabilizes G-quadruplex structures in telomeres and the c-myc promoter, supporting its anticancer potential (46).

Moreover, TP53, one of the most frequently mutated tumor suppressor genes in cancer cells, plays a crucial role in maintaining genomic integrity. Piperine has been shown to significantly inhibit the proliferation of both triple-negative breast cancer (TNBC) cells and estrogen receptor-positive breast cancer cells, regardless of their p53 status (48). Significantly, piperine-mediated growth inhibition does not require functional p53, making it a promising agent for targeting cancers in which p53 is frequently mutated and non-functional (31). *In vivo* studies provide additional evidence supporting piperine’s role in targeting genomic instability. In a rat model of N-nitroso-N-methylurea-induced mammary tumorigenesis, piperine significantly inhibited tumor growth while downregulating key oncogenic regulators, including E-cadherin (E-cad), estrogen receptor (ER), matrix metalloproteinases (MMP-2 and MMP-9), vascular endothelial growth factor (VEGF), and c-Myc—factors that contribute to genetic alterations and cancer progression (49).

### 4.2 Role of piperine in sustained proliferative signaling

Healthy cells require mitogenic growth signals to transition into an actively proliferative state. These signals include diffusible growth factors (GFs), extracellular matrix (ECM) components, and molecules that maintain cell adhesion (50). These inductive signals guide the process of cell division, producing daughter cells. In normal tissues, the production and release of these growth signals are tightly regulated to maintain cellular homeostasis, ensuring both structural integrity and functional stability (51).

In healthy cells, these signals are transient; however, in cancerous cells, they persist chronically. This persistent signaling leads to mutational changes in genes within cancer cells, known as oncogenes, which drive cell proliferation (52). It has also been discovered that cancer cells can become self-sufficient, enabling these signals to continuously maintain their proliferative pathways. Consequently, cancer cells can induce neighboring cells to support their growth by producing numerous growth factors (GFs). Furthermore, cancer cells can increase the number of transmembrane receptors, resulting in an enhanced response to growth factors and an increased proliferation rate. These receptors possess intracellular tyrosine kinase activity, such as the epidermal growth factor receptor (EGF-R/erbB) found in breast, brain, and stomach tumors (53). To inhibit sustained proliferation in cancer, several critical signaling pathways serve as primary targets, including hypoxia-inducible factor-1 (HIF-1), NF-κB, PI3K/Akt, insulin-like growth factor receptor (IGF-1R), cyclin-dependent kinases (CDKs), and estrogen receptor signaling (53).

Piperine exhibits considerable antiproliferative effects across various cancer cell types by inducing cell-cycle arrest at different phases (G0/G1, S, or G2/M), depending on the cancer type and classification (8). For example, in SKMEL-28 and B16F0 melanoma cells, piperine promotes G1 phase arrest by downregulating cyclin D1 and upregulating p21 (54). This effect is potentially regulated by ROS-induced DNA damage, as demonstrated by H2AX phosphorylation at Ser139, which in turn activates ATR and Chk1, leading to cell cycle arrest and apoptosis (54). Piperine also activates DNA damage response pathways (e.g., ATR/Chk1/p53/p21 axis), resulting in further cell-cycle arrest and apoptosis. In prostate cancer cells (PC3, LNCaP, and DU145), piperine arrests the cell cycle at the G0/G1 phase through downregulation of cyclins D1 and A, accompanied by upregulation of p21 and p27 proteins. However, the arrest was noticeably less pronounced in PC3 cells due to weaker p21 and p27 induction (55). This phase arrest is associated with the downregulation of cyclin D and the upregulation of p21. As an inhibitor of cyclin-dependent kinases (CDKs), p21 prevents phosphorylation of the retinoblastoma protein (pRb) and reduces the expression of E2F transcription factor 1 (E2F1), thereby hindering progression of the cell cycle into and through the S phase (8).

Furthermore, piperine inhibits voltage-gated K^+^ channels in prostate cancer cells, contributing to G0/G1 phase arrest and apoptosis (8). It has also been observed that piperine induces cell cycle arrest in the S phase in leukemia cells (56). Additionally, piperine promotes G2/M phase arrest in osteosarcoma cells, characterized by the downregulation of cyclin B1, CDK1, and Cdc25C, along with increased phosphorylation of CDK1 and Chk2 (57). DNA damage induced by reactive oxygen species (ROS), as evidenced by H2AX phosphorylation, further contributes to apoptosis. Moreover, Jain and colleagues demonstrated that piperine elevates intracellular ROS levels in HeLa (cervical cancer) and MCF-7 (breast cancer) cells, leading to G2/M phase cell cycle arrest and a subsequent decrease in cell viability (58).

In colon cancer cells, piperine at concentrations of 75–150 μM induces G1 phase cell cycle arrest by downregulating cyclin D1, cyclin D3, CDK4, and CDK6, thereby preventing uncontrolled proliferation. Similarly, in osteosarcoma and triple-negative breast cancer (TNBC) cells, piperine induces G2/M phase arrest, suppresses proteins involved in cell cycle progression, and enhances the expression of the cyclin-dependent kinase inhibitor p21 (Waf1/Cip1). In a study conducted by Sriwiriyajan et al., piperine demonstrated a significant inhibitory effect on proliferation in rats with N-nitroso-N-methylurea-induced mammary tumorigenesis. This effect was associated with the downregulation of E-cadherin (E-cad), estrogen receptor (ER), matrix metalloproteinases 2 and 9 (MMP-2 and MMP-9), vascular endothelial growth factor (VEGF), and c-Myc, while concurrently upregulating p53 (59, 60). Overall, piperine’s modulation of cell cycle regulators underpins its potent antiproliferative and pro-apoptotic effects in various cancer cells.

### 4.3 Role of piperine in inhibiting angiogenesis

Tumors require a continuous supply of oxygen, glucose, and nutrients, as well as efficient waste removal, similar to normal organs. This is facilitated by angiogenesis, the formation of new blood vessels, which is often persistently activated in tumors (45). When tumor cells are located more than 200 μM from the nearest capillary, they experience stress and activate hypoxia-inducible factors (HIF), which induce the expression of genes that promote angiogenesis (61). However, tumor blood vessels are typically abnormal, resulting in poor circulation, hypoxia, and acidosis within the tumor microenvironment. These conditions can impede treatment efficacy and promote drug resistance. Some tumors may also invade adjacent normal tissues and blood vessels, bypassing the need for angiogenesis (52). During malignancy, an “angiogenic switch” is activated in tumor cells, triggering the production of angiogenic factors that promote vascularization, thereby supplying dividing cancer cells with the oxygen and nutrients necessary for proliferation (62).

Piperine has been shown to regulate multiple aspects of angiogenesis by inhibiting proliferation, migration, and tube formation in human umbilical vein endothelial cells (HUVECs), which serve as a key model for studying angiogenesis (63). Additionally, piperine is reported to suppress collagen-induced angiogenesis in rat aorta ring explants and breast cancer cell-induced angiogenic activity in chick embryos (64).

At the molecular level, piperine inhibits Akt phosphorylation at Ser473 and Thr308, leading to the suppression of the PI3K/Akt signaling pathway, which is crucial for endothelial cell function and angiogenesis (64). Vascular endothelial growth factor (VEGF) is a significant activator of the PI3K/Akt pathway in endothelial cells (65). Inhibition of this pathway through the use of piperine results in the suppression of endothelial cell migration. Piperine has been observed to reduce VEGF expression in a dose-dependent manner at optimal doses, further confirming its role in inhibiting tumor-promoting angiogenesis (49). Additionally, piperine, in the form of *P. longum*, inhibits VEGF, pro-inflammatory cytokines, and melanoma cell-induced angiogenesis in C57BL/6 mice (66).

The tumor microenvironment—which includes extracellular matrix (ECM) molecules, tumor cells, endothelial cells, cancer-associated fibroblasts, and immune-responsive cells—plays a critical role in regulating angiogenesis. Piperine has been shown to modulate the tumor microenvironment, indicating its potential for cancer prevention and treatment (67). Furthermore, studies demonstrate that piperine downregulates MMP-9 and VEGF mRNA expression while upregulating E-cadherin, a key adhesion molecule essential for maintaining ECM integrity, thereby supporting its anti-metastatic properties (68). Piperine’s ability to suppress angiogenic factors, inhibit VEGF expression, and impair endothelial cell function underscores its promising role as an anti-angiogenic and chemopreventive agent in cancer therapy (49, 64, 68).

### 4.4 Role of piperine in inhibiting metastasis

A defining characteristic of advanced cancers is their ability to invade surrounding tissues and metastasize. Cancer cells accomplish this by infiltrating local tissues and entering the blood and lymphatic vessels to spread throughout the body.

Metastasis involves the transport of cancer cells to lymph nodes and distant organs, where new tumors can develop. This process begins with the disruption of cell–cell adhesion structures, including tight junctions, adherens junctions, gap junctions, desmosomes, and hemidesmosomes (49). Metastasis is regulated by intrinsic cancer cell programs, notably the epithelial-mesenchymal transition (EMT), as well as external signals from the tumor microenvironment, such as the hypoxia response system mediated by HIF proteins. These mechanisms control the expression of genes that facilitate cell migration, survival in circulation, and growth at secondary sites (52, 69). The ability to invade and metastasize can arise either early or late in cancer progression and may be considered either a selected advantage or an unintended consequence of other activated pathways (52).

Piperine exhibits anti-metastatic properties by inhibiting key pathways involved in cancer cell invasion and migration. In a murine lung metastasis model, piperine reduced tumor nodule size and decreased levels of uronic acid, hexosamine, and hydroxyproline, indicating its role in preventing metastasis (10). In gastric cancer cells, it suppressed IL-6 via the c-Src/RhoA/ROCK signaling pathway, thereby inhibiting cancer cell invasion and metastasis (68).

Piperine further inhibited the growth and metastasis in a 4T1 mouse breast cancer model. When administered intratumorally at doses of 2.5 and 5 mg/kg every three days, piperine significantly suppressed primary tumor growth *in vivo*, with the higher dose also reducing lung metastasis. These results, observed in a highly aggressive breast cancer model, underscore piperine’s potential to both inhibit tumor progression and prevent metastatic spread (69). It reduced the expression of MMP-2 and MMP-9 in breast cancer cells, partly through the downregulation of HER2, which is critical for metastatic potential (70). The inhibition of MMPs was linked to the suppression of Akt signaling in breast, osteosarcoma, prostate, and fibrosarcoma cancer cells (68, 71). Similarly, in mouse mammary carcinoma cells, piperine suppressed MMP-13, which is frequently expressed in invasive breast cancer (8). Additionally, piperine increased the expression of TIMP-1 and TIMP-2 in osteosarcoma cells, counteracting MMP-2 and MMP-9 activity and thereby inhibiting metastasis development (57). Piperine inhibited the migration of prostate, stomach, and colon cancer cells by downregulating STAT-3, highlighting its role in suppressing metastasis (72). Additionally, it targeted cancer cell migration-related epithelial-mesenchymal transition (EMT). Piperine inhibited TGF-β1-induced EMT in A549 lung cancer cells by restoring epithelial markers, suppressing mesenchymal markers, and reducing MMP expression (73, 74). In colon cancer models, piperine decreased STAT-3 phosphorylation and snail expression, increased E-cadherin levels, and decreased vimentin expression, thereby preventing EMT-driven metastasis (75). Recent research has demonstrated that piperine inhibits Wnt/β-catenin signaling, a pathway associated with cancer development. In colorectal and osteosarcoma cells, piperine suppressed β-catenin and its downstream targets, including COX-2, cyclin D1, and c-myc, resulting in the induction of apoptosis and inhibition of metastasis (75, 76). Overall, piperine inhibits metastasis across various cancer types, primarily through MMP inhibition, EMT suppression, and downregulation of the Wnt/β-catenin pathway. However, further studies are needed to fully elucidate its mechanism of action.

### 4.5 Role of piperine in disrupted differentiation

Cancer stem cells (CSCs) possess self-renewal capabilities and generate diverse cancer cell populations, playing a crucial role in tumor initiation and relapse (77). Recent research shows that CSCs display high adaptability and depend on stemness pathways for resistance and self-renewal. This highlights the importance of targeting these pathways to prevent treatment failure (78). These cells exhibit altered cellular energetics, promote tumor-associated inflammation, evade apoptosis, resist immune destruction, and demonstrate high resistance to anticancer drugs (10). Several key signaling pathways regulate CSC self-renewal and differentiation, including Wnt/β-catenin, Hedgehog, Notch, JAK-STAT, NF-κB, PI3K/Akt/mTOR, and TGF/SMAD (79).

Targeting these pathways with chemopreventive agents may aid cancer prevention. Piperine may indirectly restore differentiation programs and inhibit aberrant self-renewal by modulating these stemness-associated pathways. Research on colorectal and breast cancer cells shows that piperine inhibits Wnt/β-catenin signaling (76) and also affects the PI3K/Akt/mTOR pathway (80). In cervical cancer cells, the combination of piperine with mitomycin-C suppresses STAT3/NF-κB activation, leading to inhibition of the Bcl-2 pathway (81). Piperine, for example, enhances osteoblast differentiation through AMPK phosphorylation and the upregulation of osteogenic markers (82). The Wnt/β-catenin, Hedgehog, and Notch pathways play key roles in cancer stem cell (CSC) regulation, and piperine influences all of these pathways. Specifically, piperine inhibits the Wnt/β-catenin signaling pathway in breast cancer stem cells, thereby altering their self-renewal properties (83). In addition, piperine regulates key proteins such as DKK-1, sFRP2, Bmi-1, and CDK6, maintaining a balance between proliferative and quiescent cancer stem cells (CSCs) (84). At micromolar (µM) concentrations, piperine significantly reduced both primary and serially passaged mammospheres, as well as the percentage of ALDH^+^ stem/progenitor cells, thereby inhibiting breast cancer stem cell self-renewal and mammosphere formation *in vitro* (85). *In vivo*, piperine enhanced chemotherapeutic efficacy in models of triple-negative breast cancer by reducing the cancer stem cell (CSC) marker ALDH-1 and suppressing the PI3K/Akt/mTOR signaling pathway, suggesting direct interference with stemness-associated pathways in tumors (86).

### 4.6 Role of piperine in resisting cell death

#### 4.6.1 Apoptosis

Apoptosis includes several steps: disruption of cellular membranes, breakdown of structures within the cytoplasm and nucleus, expulsion of cytosol, degradation of chromosomes, and finally, fragmentation of the nucleus. Typically, within 24 hours, adjacent cells in the tissue consume the shriveled remains, causing them to eventually disappear (87).

It is regulated by sensors such as p53 and members of the Bcl-2 family, including Bax and Bak, which detect cellular stress. When these sensors identify irreparable damage, they activate effector caspases that initiate cell destruction. Cancer cells often evade apoptosis by disabling these sensors (e.g., through p53 mutations), downregulating pro-apoptotic factors (e.g., Bax and Bak), upregulating anti-apoptotic factors (e.g., Bcl-2 and Bcl-xL), or enhancing survival signaling pathways (e.g., IGF-1 and IGF-2), thereby promoting uncontrolled proliferation (88).

Apoptosis can be initiated through two primary pathways: the mitochondrial-mediated intrinsic pathway and the death receptor-mediated extrinsic pathway. The intrinsic pathway is activated by the release of apoptogenic factors such as cytochrome c, Smac/DIABLO, and apoptosis-inducing factor (AIF) into the cytosol, leading to caspase activation (8). Numerous chemopreventive agents, including piperine, induce apoptosis via both pathways. Piperine exerts cytotoxic effects on various human cancer cell lines (MCF-7, HepG2, HeLa, and PC3), exhibiting classic apoptotic features such as nuclear condensation, phosphatidylserine externalization, and DNA fragmentation (10). Studies have shown that piperine modulates Smac/DIABLO in HER2-expressing breast cancer cells, supporting its role in the intrinsic apoptotic pathway (68). Similarly, research in melanoma cells demonstrated that piperine downregulates the anti-apoptotic protein XIAP, facilitating caspase-9 activation and apoptosome formation (89). Additional evidence from various cancer models—including melanoma, hepatocarcinoma, breast, lung, prostate, leukemia, and ovarian cancer cells—confirms that piperine promotes apoptosis by increasing Bax expression while simultaneously decreasing Bcl-2 levels (8). In prostate cancer cells, piperine induces apoptosis by cleaving PARP-1, suppressing phosphorylated STAT-3, and inhibiting NF-κB expression, ultimately reducing cell proliferation and promoting cell death. This evidence confirms piperine’s role in initiating apoptosis through caspase activation, particularly caspases-3 and -9 (90). Piperine also exhibits anticancer potential in ovarian cancer by activating the intrinsic apoptotic pathway via JNK/p38 MAPK signaling (91). Additionally, piperine downregulates the uncleaved Bid protein in melanoma cells, suggesting a potential link between the intrinsic and extrinsic apoptotic pathways, although no direct activation of caspase-8 has been observed (8). The inhibition of piperine-induced apoptosis by caspase-3 and -9 inhibitors, but not by caspase-8 inhibitors, further supports that piperine predominantly activates the intrinsic pathway (91).

Siddiqui (2017) confirmed that piperine promotes apoptosis in a dose-dependent manner through the generation of reactive oxygen species (ROS), disruption of mitochondrial membrane potential, DNA fragmentation, and activation of caspase-3 (92).

Beyond inducing apoptosis, piperine modulates several other key cancer-related pathways. It inhibits fatty acid synthase expression in HER2-overexpressing breast cancer cells by suppressing ERK1/2 signaling and reducing SREBP-1 levels, thereby promoting apoptosis. Additionally, piperine binds with high affinity to G-quadruplex structures in the c-myc promoter region in lung cancer cells, leading to decreased c-myc expression and subsequent apoptotic effects (8). Numerous *in vivo* animal studies demonstrating the anticancer activity of piperine are summarized in a comprehensive review. These studies include mouse and rat models of Ehrlich carcinoma, hepatocellular carcinoma, melanoma, gastric cancer xenografts, and other tumor types, all showing apoptosis induction and tumor growth inhibition. Although the specifics vary between models, there is consistent evidence that piperine possesses anticancer potential beyond *in vitro* systems (93).

#### 4.6.2 Autophagy

Autophagy is a cellular recycling system that degrades organelles to provide nutrients during starvation and promote cell survival. However, excessive stress can overactivate autophagy, leading to “autophagy-associated cell death” due to the excessive breakdown of organelles. This process is distinct from apoptosis and necroptosis and can either support cell survival and proliferation or contribute to cell elimination, depending on the cell’s physiological state (52). In this context, autophagy plays a crucial role in cancer by maintaining cellular homeostasis, recycling damaged organelles, and promoting tumor survival under stress conditions such as hypoxia and metabolic stress. It supports oncogenic transformation by increasing the expression of ATG5 and ATG7, which are essential for malignant cell growth. Moreover, autophagy is linked to epithelial-mesenchymal transition (EMT), a key process in cancer metastasis, through pathways involving Beclin-1, p62, and TGF-β. It also helps tumor cells resist anoikis (detachment-induced cell death) and contributes to the survival of cancer stem cells, which is associated with chemotherapy resistance.

Due to its role in tumor progression and therapy resistance, autophagy is considered a promising target for cancer treatment. Several inhibitors, including Wortmannin, Spautin-1, and hydroxychloroquine derivatives, have demonstrated significant potential in preclinical and clinical studies by blocking autophagic pathways and enhancing the efficacy of existing therapies (53).

Piperine has been implicated in the regulation of autophagy. In prostate and leukemia cancer cells, piperine was shown to induce autophagy, as evidenced by an increase in LC3B-II expression and the formation of LC3B puncta (25).

Piperine has also been observed to modulate autophagy, a process that can either promote or inhibit cancer development (10). By inhibiting mTORC kinase activity, piperine induces autophagy in cancer cells, as demonstrated by the regulation of key autophagy-related proteins, including LC3, p62, and beclin-1, in Bel-7402/5-FU cells (94). Furthermore, *in vivo* studies have shown that piperine inhibits PI3K signaling, resulting in reduced oral cancer tumor growth (10). In a CT26 colorectal cancer cells xenograft mouse model, piperine treatment significantly reduced tumor weight and volume, indicating effective suppression of tumor growth *in vivo*. This effect was associated with the inhibition of signaling pathways involved in cancer progression and the induction of autophagy (95). Another study evaluated the effects of piperine in a nude mouse xenograft model and on HSC-3 oral cancer cells. Daily administration of piperine (50 mg/kg) for four weeks significantly reduced tumor weight and volume. Mechanistic analysis supported piperine’s therapeutic potential against oral cancer *in vivo*, revealing that it induced autophagy and apoptosis by inhibiting the PI3K/Akt/mTOR pathway (25).

#### 4.6.3 Ferroptosis

Iron-dependent lipid peroxidation and the catastrophic disruption of redox homeostasis are hallmarks of ferroptosis, a regulated, non-apoptotic form of cell death (96). Unlike apoptosis, ferroptosis is driven by the accumulation of lipid reactive oxygen species (ROS), depletion of intracellular glutathione (GSH), and functional inactivation of glutathione peroxidase 4 (GPX4), a critical enzyme that protects cells from membrane damage caused by lipid peroxides (97, 98). This pathway represents a promising therapeutic target, particularly in tumors resistant to apoptosis, because dysregulated iron metabolism, increased ROS production, and impaired antioxidant defenses render cancer cells especially vulnerable to ferroptotic death.

According to previous research, piperine’s ability to disrupt redox balance and induce oxidative stress in cancer cells may enhance ferroptosis-related signaling. Piperine treatment has been shown to significantly increase intracellular reactive oxygen species (ROS) levels and lipid peroxidation in various cancer models, leading to oxidative damage and suppression of tumor growth *in vivo* (73, 99). Piperine’s antitumor efficacy was significantly diminished by antioxidant co-treatment in a diethylnitrosamine-induced hepatocellular carcinoma rat model, providing *in vivo* evidence that its anticancer activity is mediated, at least in part, through a pro-oxidant mechanism (73). Ferroptotic cell death, which depends on reactive oxygen species (ROS) accumulation and lipid peroxide overload, aligns mechanistically with these redox-disruptive effects.

Current *in vivo* and mechanistic data support the hypothesis that piperine may sensitize cancer cells to ferroptosis-like cell death, although direct confirmation of canonical ferroptosis markers, such as GPX4 inhibition, system Xc^-^ suppression, or iron dependency, remains limited. To conclusively demonstrate that this pathway contributes to piperine’s anticancer effects, further research employing ferroptosis-specific inhibitors and validated molecular markers is required.

#### 4.6.4 Anoikis

Anoikis is a specific form of apoptosis that occurs when cells detach from the extracellular matrix (ECM). It serves as a crucial defense mechanism that prevents detached epithelial cells from proliferating in inappropriate locations (100). However, in cancer, tumor cells often develop resistance to anoikis, enabling them to proliferate, invade distant organs, and form metastases. This resistance is frequently mediated by the activation of the FAK/PI3K/Akt and mTOR signaling pathways, alterations in integrin expression, and enhanced antioxidant defenses, which collectively allow cells to evade detachment-induced apoptosis (101).

Emerging reports indicate that piperine can counteract anoikis resistance by inhibiting the FAK/PI3K/Akt/mTOR pathway and increasing reactive oxygen species (ROS) levels in cancer cells, thereby restoring sensitivity to anoikis-mediated cell death (25).

Additionally, Anoikis can be induced in melanoma cells *in vitro* by piperlongumine, a structural analogue of piperine, through the suppression of STAT3 (54). To fully elucidate piperine’s role in promoting anoikis-mediated tumor suppression, further mechanistic and *in vivo* studies are required.

### 4.7 Role of piperine in inflammation

Developing cancers exploit inflammation, characterized by the infiltration of immune cells (IIC), to acquire key cancer hallmarks. Although inflammation is typically a defensive response, IIC paradoxically promotes tumor progression. Inflammation enhances multiple cancer hallmarks by supplying growth factors that support proliferative signaling and survival. It also obstructs cell death through the release of proangiogenic factors and extracellular matrix-modifying enzymes that facilitate angiogenesis, invasion, and metastasis, as well as signals that activate epithelial-mesenchymal transition (EMT) and other hallmark-enabling processes (52). Chronic inflammation is strongly associated with cancer progression, with cytokines such as interleukins, TNF-α, TGF-β, and granulocyte-macrophage colony-stimulating factor, along with chemokines and transcription factors including NF-κB, STAT3, and HIF-1α, playing crucial roles in cancer-related inflammation (62, 102). Notably, inflammation is commonly observed during early neoplastic progression and stimulates the transformation of precancerous lesions into malignant tumors (103). Furthermore, inflammatory cells release reactive oxygen species that induce mutations in cancer cells, accelerating their evolution toward more aggressive phenotypes (104).

Piperine has emerged as a promising anti-inflammatory agent for cancer prevention and therapy. Various *in vitro* studies have evaluated its ability to regulate inflammation using different isolated cell models (105). For example, interferon-gamma (IFN-γ) production has been assessed in human peripheral blood mononuclear cells (PBMCs) (106). In HaCaT keratinocyte cells exposed to UV-B-induced oxidative stress, piperine reduced the production of reactive oxygen and nitrogen species (ROS/RNS), leading to decreased expression of inflammatory mediators such as p38, JNK, AP-1, iNOS, and COX-2 (105). Piperine also demonstrated significant anti-inflammatory activity in rat models of hind paw swelling and arthritis. Additionally, piperine administration reduced histological damage and myeloperoxidase activity in the pancreas, as well as improved parameters, including the pancreatic weight-to-body weight ratio and serum levels of amylase, lipase, and trypsin activity. Studies involving piperine pretreatment have shown that it lowers the production of tumor necrosis factor (TNF)-α, interleukin (IL)-1β, and IL-6 during cerulein-induced acute pancreatitis (AP). *In vivo* results further support the conclusion that piperine reduces cell death, amylase and lipase activities, and cytokine production in isolated cerulein-treated pancreatic acinar cells. Moreover, piperine inhibits the activation of mitogen-activated protein kinases (11).

### 4.8 Role of piperine in immune system modulation

Cancer tumors must evade immune detection to ensure their survival and proliferation. A robust immune system can effectively eliminate highly immunogenic tumors through a potent innate immune response. However, when the immune system is compromised or the tumor exhibits reduced immunogenicity, the elimination process may be incomplete. This leads to a state of equilibrium, in which the tumor persists under immune surveillance (52). This equilibrium is characterized by a dynamic interplay between tumor growth and immune-mediated destruction, resulting in alternating phases of slow tumor expansion, immune-driven elimination, and subsequent regrowth. This balance can last a lifetime or be disrupted, allowing the tumor to escape immune control. Approximately 20% of human tumors induced by viruses exhibit distinct immune responses (49). Cancer cells employ various strategies to evade immune surveillance, including modulation of immune checkpoint pathways and recruitment of immunosuppressive cells such as regulatory T cells and myeloid-derived suppressor cells. Additionally, they impair components of the immune system by suppressing cytotoxic T lymphocytes (CTLs) and natural killer (NK) cells through the overexpression of transforming growth factor-beta (TGF-β) and other immunosuppressive factors (52).

Increased neutrophil levels are associated with a reduction in lymphocytes and impaired immune function (107). The researchers observed that piperine from *Piper nigrum* extract (PFPE) significantly decreased neutrophil counts while increasing lymphocyte levels compared to the control group. This reduction in neutrophils may be attributed to the D-limonene compound in PFPE, which has been shown to lower neutrophil counts in immunocompromised mice (108). T lymphocytes, the primary cells of the adaptive immune system, play a crucial role in eliminating infected and foreign cells, activating other immune cells, secreting cytokines, and regulating the cancer immune response (109). β-Caryophyllene, a major compound in PFPE, exerts anti-inflammatory effects by modulating the secretion of Th1/Th2 cytokines (110). Additionally, all doses of PFPE significantly increased T helper type 1 (Th1) cells while decreasing T helper type 2 (Th2) and regulatory T (Treg) cells. However, the precise mechanism by which PFPE modulates T lymphocytes remains unclear and warrants further investigation. Piperine exhibited immunomodulatory effects on tumor-associated inflammation in a cervical cancer model by suppressing tumor growth *in vivo*, downregulating pro-inflammatory cytokines (IL-1β, IL-17A, IL-21, and IL-22), and reducing the number of Th17 cells (111).

### 4.9 Piperine disrupts redox homeostasis in cancer

Redox homeostasis is the balance between the production and elimination of reactive oxygen species (ROS) and other free radicals. It is believed that cancer cells exhibit an abnormal redox status, characterized by increased baseline production of reactive oxygen species. This elevated ROS level makes cancer cells more susceptible to disruptions in oxidative stress balance, which can lead to cellular damage (112, 113).

Piperine plays a dual role in redox homeostasis, functioning as both an antioxidant and a pro-oxidant depending on its concentration and the cellular context. Reactive oxygen species (ROS) and free radicals, produced by enzymatic sources as well as non-enzymatic activity in the mitochondrial electron transport chain, contribute significantly to cancer development (114–116).

Piperine influences the redox balance in cancer cells by modulating reactive oxygen species (ROS) levels. At low concentrations, it functions as a potent antioxidant, scavenging free radicals and reactive metabolic intermediates, thereby reducing oxidative stress and inhibiting tumorigenesis (117).

Moreover, at higher concentrations, piperine acts as a pro-oxidant, disrupting redox homeostasis and inducing oxidative stress-mediated apoptosis in cancer cells. Cancer cells inherently produce elevated levels of reactive oxygen species (ROS), which promote oncogenic signaling and tumor progression (115). This heightened oxidative state makes them particularly susceptible to pro-oxidant agents such as piperine, which further increases ROS production beyond tolerable thresholds, leading to cytotoxicity (118). Piperine has been shown to activate the radical-mediated mitochondrial apoptotic pathway in hepatocellular carcinoma cells (73). It stimulates ROS generation in various cancer types, including HeLa and MCF7 cells, especially when delivered via nanofiber mats (58). Additionally, piperine induces ROS-dependent dissipation of mitochondrial membrane potential, caspase activation, and cell cycle arrest in human oral squamous carcinoma cells (92) and HRT-18 rectal adenocarcinoma cells (119).

Moreover, in PC3 human prostate cancer cells, piperine exposure increased intracellular reactive oxygen species (ROS) and calcium levels, leading to mitochondrial membrane depolarization and programmed cell death (58). Another study demonstrated that piperine significantly improved liver histology and inhibited the progression of diethylnitrosamine (DEN)-induced hepatocarcinoma in rats. Importantly, co-treatment with the antioxidant EUK-134 markedly reduced piperine’s anticancer effects, indicating that pro-oxidant activity and redox disruption are integral to its *in vivo* anticancer mechanism (8).

In essence, piperine’s ability to modulate redox homeostasis in a concentration and context-dependent manner, shifting from an antioxidant at low levels to a pro-oxidant at higher levels that exacerbates the inherent oxidative stress in cancer cells, underscores its potential as a targeted anti-cancer agent by disrupting reactive oxygen species (ROS) balance and inducing cell death pathways. The mechanisms through which piperine exerts anticancer activity are summarized in **Table 1**.

**Table 1:** The multifaceted mechanisms through which piperine exerts anticancer activity across the hallmarks of cancer.

Cancer hallmark	Molecular target(s)/pathway(s)	Mechanism of action	Outcome	Limitations of evidence	Reference
Sustaining Proliferative Signaling	Cyclin D1, CDKs, p21, p27, ATR/Chk1, ROS	Induces cell cycle arrest (G0/G1, G2/M); upregulates CDK inhibitors	Inhibits uncontrolled proliferation	The diversity of experimental paradigms and the poor mechanistic integration across signaling pathways.	(8, 54, 55, 57–60)
Genomic Instability	G-quadruplexes, telomerase, c-Myc, p53-independent pathways	Stabilizes G-quadruplex DNA; downregulates telomerase and c-Myc	Promotes genomic integrity	The majority of research is still theoretical or mechanistic, and there is little experimental support for its wider influence on genomic instability in cancer.	(31, 41, 46–49)
Evading Growth Suppressors	p53, pRb, TGF-β, E2F1	Modulates p53 and Rb-related control pathways	Restores cell cycle checkpoints	Few studies; conclusions are based largely on secondary literature summarizing *in vitro* and *in vivo* studies. Clinical evidence remains limited.	(8, 82)
Enabling Replicative Immortality	Telomerase, c-Myc	Inhibits telomerase via G-quadruplex stabilization	Limits indefinite replication	Some evidence is derived from biochemical DNA interaction studies and investigations on piperine analogs, which do not fully represent the activity of native piperine.	(41, 46, 47)
Resisting Cell Death (Apoptosis)	Bcl-2, Bax, Caspases, PARP, ROS	Triggers intrinsic apoptotic pathways; increases Bax, caspase-3/9, ROS	Induces programmed cell death	Most data originate from cell-based studies across various cancer types, with limited translational validation.	(8, 10, 68, 89–93)
Autophagy	mTOR, LC3B, p62, Beclin-1	Regulates autophagy via mTOR inhibition and upregulation of LC3B and beclin-1	Promotes or inhibits tumor survival contextually	The results are based on mechanistic investigations in particular cell models and piperine derivatives, which restricts their generalizability.	(10, 25, 94, 95)
Ferroptosis	ROS, Ca²^+^, GSH, lipid peroxides	Enhances oxidative stress and iron-dependent cell death	Induces ferroptosis in cancer cells	There is little direct evidence of ferroptosis in tumor models, and conclusions are based on reviews and indirect preclinical data.	(73, 99)
Reprogramming Metabolism	GLUT transporters, PKM2, HK2	Alters glucose uptake and glycolytic enzyme activity	Disrupts energy metabolism	Incomplete assessment of metabolic reprogramming *in vivo* and mechanistic pathways.	(82, 90, 119),
Immune Evasion	Tregs, Th1/Th2 ratio, β-caryophyllene, STAT3	Enhances immune surveillance; reduces immunosuppressive cells	Promotes immune-mediated tumor clearance	Evidence includes *in vitro*, *ex vivo*, and computational studies with limited direct validation of piperine’s effects on tumor immune modulation *in vivo.*	(107–111)
Tumor-Promoting Inflammation	NF-κB, COX-2, IL-1β, IL-6, TNF-α	Suppresses inflammatory cytokines and transcription factors	Reduces inflammation-mediated tumor progression	Limited direct evaluation in tumor-associated inflammation.	(11, 105, 106)
Angiogenesis	VEGF, Akt, PI3K, HUVEC migration	Inhibits VEGF expression and endothelial cell activity	Reduces blood vessel formation	Few direct *in vivo* studies have validated the antiangiogenic effect in tumors.	(63–68)
Invasion and Metastasis	MMP-2/9/13, TIMP-1/2, HER2, EMT markers (E-cadherin, vimentin, Snail, STAT3)	Suppresses EMT, downregulates MMPs, blocks STAT3, and HER2	Inhibits cancer spread	Inadequate clinical validation and a narrow assessment of various cancer types and molecular pathways.	(8, 10, 57, 68–76),
Cancer Stem Cell Regulation	Wnt/β-catenin, Notch, PI3K/Akt/mTOR, CDK6, ALDH-1	Blocks self-renewal signaling and mammosphere formation	Limits recurrence and resistance	No thorough studies have investigated its long-term effects on tumor relapse, cancer stem cell survival, and plasticity.	(76, 80–86)
Redox Homeostasis	ROS, MMP, antioxidant enzymes	Acts as an antioxidant at low doses; pro-oxidant at high doses	Induces selective cancer cell toxicity	The exact molecular processes controlling redox homeostasis and the equilibrium between its pro-oxidant and antioxidant activities are still not well understood.	(8, 58, 73, 92, 114–119)

## 5 Piperine formulation

### 5.1 Piperine-loaded PLGA nanoparticles

Piperine’s poor aqueous solubility, low bioavailability, and quick metabolic degradation limit its clinical translation despite its promising properties. Poly (lactic-co-glycolic acid) (PLGA)-based nanoparticle systems have been extensively investigated as an efficient piperine delivery system in cancer therapy to overcome these constraints.

The successful production of piperine-loaded PLGA nanoparticles with an average particle size of roughly 95 nm and a negative surface charge, which are advantageous for cellular uptake and systemic stability, was shown in one of the most thorough investigations. The drug release profile of the nanoparticles was pH-responsive and sustained; under acidic conditions (pH 5.5), which mimic the tumor microenvironment, the release was noticeably higher. *In vitro* experiments showed decreased toxicity toward normal HEK-293 cells and increased cytotoxicity against several cancer cell lines, such as HeLa, A549, PC-3, and MCF-7 cells. The formulation also successfully prevented cancer cells from migrating, suggesting that it may have antimetastatic properties (120).

Further developments with PEGylated PLGA (PLGA-b-PEG) nanoparticles encapsulating piperine to enhance circulation time and colloidal stability were documented. Compared with free piperine, these nanoparticles exhibited noticeably greater cytotoxicity in human colorectal carcinoma (HCT116) cells. They had sizes between 44 and 49 nm and a high encapsulation efficiency of about 80%. The nanoformulation demonstrated enhanced intracellular accumulation, induced dose- and time-dependent apoptosis, and promoted cell cycle arrest in the G0/G1 phase, underscoring its potential for colorectal cancer treatment (121).

**Figure 5:**
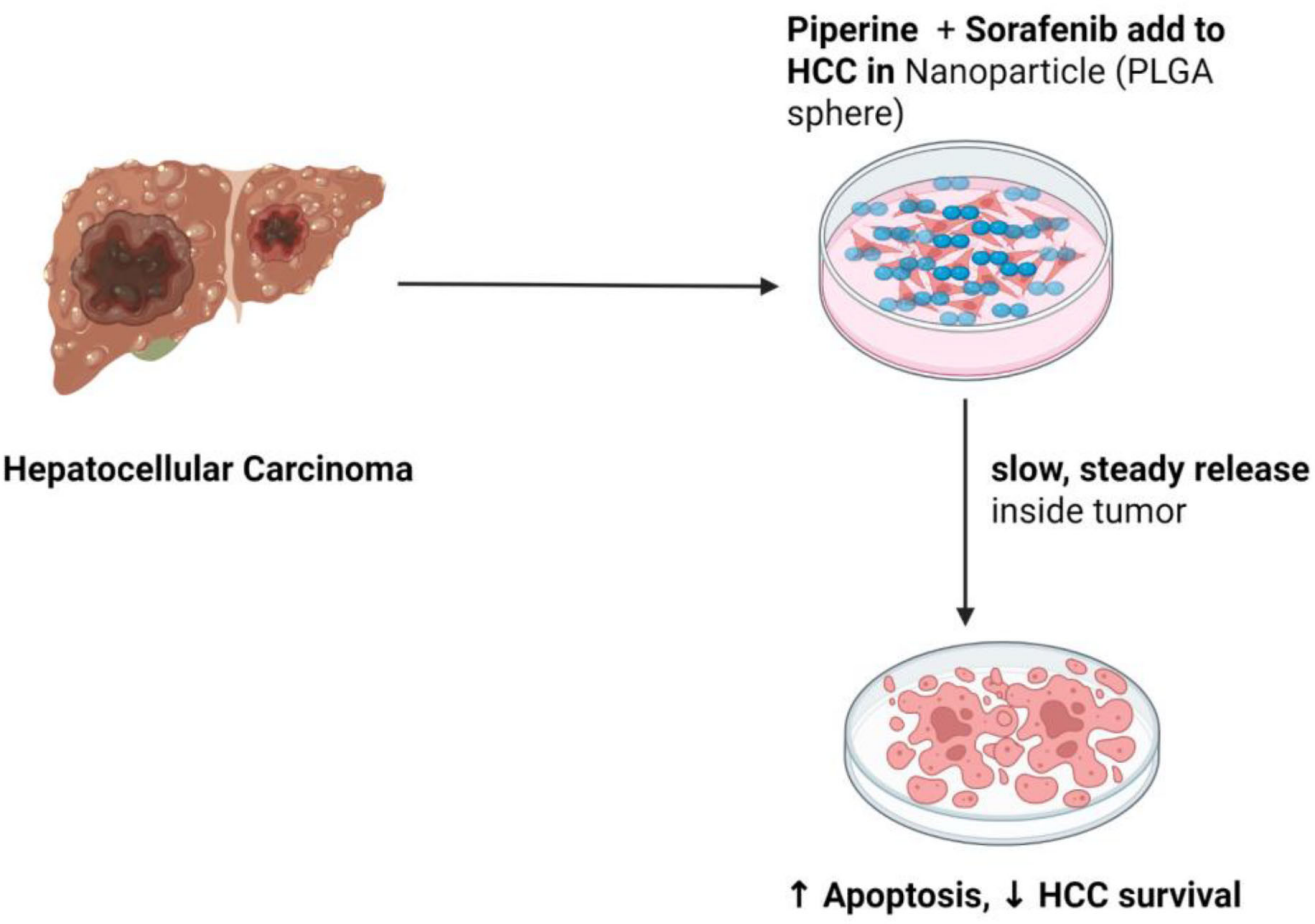
Piperine-based nanoparticle strategy in hepatocellular carcinoma. The figure was created using BioRender.com.

Additionally, piperine has demonstrated potent anticancer activity against hepatocellular carcinoma (HCC) cells and has been shown to improve the pharmacokinetic (PK) properties of several anticancer drugs, including sorafenib. A study presents a novel strategy to enhance HCC treatment by co-delivering piperine (PIP) and sorafenib (SORA), two poorly water-soluble drugs, using small, biodegradable poly (lactic-co-glycolic acid) (PLGA) nanoparticles (SP-PNPs). Stabilization was achieved by varying concentrations of polyvinyl alcohol (PVA). These nanoparticles were thoroughly characterized and evaluated *in vitro*. Results indicated that both drugs were successfully encapsulated within the nanoparticles in a non-crystalline form. SP-PNPs with lower concentrations of stabilizer (PVA) exhibited slow and sustained release of SORA and PIP. Furthermore, the SP-PNPs demonstrated significantly enhanced cytotoxicity against HCC cells *in vitro* compared to sorafenib alone. These findings suggest that co-delivery of PIP and SORA via nanoparticles may represent a promising approach to improve HCC treatment efficacy and warrant further investigation in animal models (122) (**Figure 5**).

### 5.2 Piperine enhances doxorubicin sensitivity in triple-negative breast cancer

Triple-negative breast cancer (TNBC) is a challenging malignancy with limited treatment options beyond standard chemotherapy, often resulting in drug resistance.

Piperine has been shown to increase triple-negative breast cancer cells’ susceptibility to chemotherapeutic drugs like doxorubicin by modifying apoptotic pathways and preventing the growth and migration of tumor cells (68).

Research indicates that the PI3K/Akt/mTOR pathway contributes to this resistance by promoting the growth of cancer stem cells (CSCs). A previous study investigated whether piperine (PIP) could sensitize TNBC cells to the chemotherapy drug doxorubicin (DOX).

Laboratory experiments on triple-negative breast cancer (TNBC) cells demonstrated that combining doxorubicin (DOX) and piperine (PIP) significantly enhanced the cytotoxicity of cancer cells compared to DOX alone. This combination more effectively inhibited the PI3K/Akt/mTOR signaling pathway, increased PTEN activity, a pathway inhibitor, and reduced ALDH-1 expression, a marker associated with cancer stem cells (CSCs).

Animal studies using a solid tumor model also supported these findings. The combination of doxorubicin (DOX) and piperine (PIP) resulted in increased tumor cell death (necrosis), decreased PTEN levels, reduced activity of key proteins in the PI3K/Akt/mTOR pathway, and lower ALDH-1 expression. In conclusion, this research suggests that adding piperine to doxorubicin treatment may help overcome drug resistance in triple-negative breast cancer (TNBC), likely by disrupting the PI3K/Akt/mTOR pathway and reducing the population of cancer stem cells (86). Similarly, piperine reduced the growth and motility of TNBC cell lines by causing cell cycle arrest, upregulating the expression of the cyclin-dependent kinase inhibitor p21, blocking Akt signaling, and initiating caspase-dependent apoptosis. These antiproliferative and pro-apoptotic effects likely sensitize TNBC cells to cytotoxic agents such as doxorubicin (68). According to another study, piperine co-delivery increases intracellular accumulation of chemotherapeutics by blocking P-glycoprotein (P-gp), which is a significant factor in TNBC multidrug resistance. This mechanism may be relevant to DOX sensitization (123).

### 5.3 Nanogel delivery loaded with curcumin and piperine potentiates glioblastoma treatment

Malignant glioblastoma (GBM), a common and aggressive brain tumor, is notably resistant to standard chemotherapy. To address this challenge, a novel gold-anchored lignin nanogel, specifically, a Lignin-g-p (NIPAM-co-DMAEMA) gold nanogel, was developed to co-deliver curcumin and piperine for the treatment of aggressive brain cancer. These nanoscale carriers enhanced drug delivery efficiency, increased cancer cell cytotoxicity, and entered cells via endocytosis, inducing programmed cell death (apoptosis) through caspase-3 activation. The combined delivery of curcumin and piperine within the nanogel demonstrated significantly superior anticancer effects compared to the individual drugs, indicating a promising new strategy to overcome the limitations of current glioblastoma therapies (124).

### 5.4 iRGD-liposomes enhance the antitumor activity of curcumin and piperine

Lung cancer is a common global malignancy with a poor survival rate despite advances in treatment, highlighting the urgent need for new, less toxic therapies. This study developed a targeted delivery system (iRGD-LP-CUR-PIP) using liposomes modified with the tumor-penetrating peptide iRGD to co-deliver curcumin (CUR) and piperine (PIP). This system was designed to enhance the targeting and uptake of therapeutic agents by lung cancer cells (125).

Lab tests demonstrated that iRGD-LP-CUR-PIP was effectively taken up by cancer cells, inhibited their growth and spread, and entered cells via an energy-independent process. *In vivo*, this targeted delivery system significantly reduced tumor size and improved overall health indicators. The iRGD modification enhanced the liposomes’ ability to target and penetrate tumors, resulting in increased drug uptake by cancer cells. The combination of CUR and PIP produced a synergistic anticancer effect. This study presents a promising targeted treatment approach using natural compounds to improve lung cancer therapy (125).

Another study showed that in non-small cell lung cancer (NSCLC) models, iRGD-LP-CUR-PIP binds to αvβ3 integrin on tumor or endothelial cells and inhibits angiogenesis by downregulating VEGF/VEGFR2 signaling and the downstream P38/MK2 pathway (126).

### 5.5 Reversing drug resistance using curcumin and piperine delivered via nanoparticles

Multidrug resistance (MDR), primarily driven by the overexpression of P-glycoprotein (P-gp), which actively pumps drugs out of cancer cells, poses a significant challenge in cancer chemotherapy. In this study, a novel co-delivery system was developed to overcome MDR. This system combined curcumin (Cur) and piperine (Pip) encapsulated within solid lipid nanoparticles (SLNs), along with tocopheryl polyethylene glycol succinate (TPGS) and Brij 78. Both TPGS and Brij 78 inhibit the P-gp drug efflux mechanism, thereby sensitizing resistant tumors. The co-delivery of Cur and Pip via SLNs significantly enhanced cytotoxicity and ensured efficient drug delivery to drug-resistant A2780/Taxol cells. This strategy shows promising potential for managing MDR in cancer treatment (127).

### 5.6 Piperine nanoparticle formulation: *in vitro* and *in vivo* evaluation for breast cancer

Piperine’s clinical application is limited by poor water solubility. To enhance piperine delivery, lipid-polymer hybrid nanoparticles (PPN-LPHNPs) were developed. The final formulation was optimized using a Box–Behnken design, focusing on drug release, cytotoxicity against breast cancer cell lines (MDA-MB-231 and MCF-7), and stability under gastrointestinal and colloidal conditions. The nanoparticle formulation demonstrated greater cytotoxicity toward cancer cells compared to free PPN. *In vivo* and *ex vivo* studies revealed increased intestinal absorption and oral bioavailability. These findings suggest that PPN-LPHNPs could improve piperine distribution and enhance its anticancer efficacy (128).

### 5.7 Micelles loaded with piperine

To enhance both water solubility and anticancer efficacy, a study aims to develop Soluplus^®^ and D-α-tocopherol polyethylene glycol succinate (TPGS) micelles. Piperine-loaded Soluplus^®^/TPGS mixed micelles demonstrated sustained drug release and outperformed free piperine *in vitro*. The MTT assay showed that these micelles were more effective against A549 and HepG2 cancer cell lines. Pharmacokinetic studies revealed that the area under the curve (AUC) for the micelle formulation was 2.56 times greater than that of free piperine. According to the study, piperine-encapsulated mixed micelles represent a promising nanocarrier for cancer chemotherapy. Delivery of piperine using Soluplus^®^/TPGS-based micellar technology proved effective in cancer treatment (129).

## 6 Clinical studies of piperine’s anticancer effects

Numerous studies have demonstrated the pharmacological effects of piperine in various diseases, prompting clinical trials to investigate these activities. Some trials have yielded promising results, confirming piperine’s efficacy in conditions such as vitiligo (15), non-alcoholic fatty liver disease (NAFLD) (130), epilepsy (131), and osteoarthritis (13). Although preclinical studies suggest that piperine has potential as an anticancer agent, more comprehensive human clinical trials are necessary to optimize its efficacy and safety as an antitumor treatment (132). Most research has focused on the effects of piperine in combination with other agents, such as curcumin (133). For example, a phase II clinical trial evaluated a combination of piperine, curcumin, and taurine—each at specific concentrations—formulated into a single capsule and administered to patients with hepatocellular carcinoma (HCC). The results indicated a potential immunostimulatory effect in these patients. Additionally, recent clinical trials have explored piperine’s role in enhancing the therapeutic efficacy of approved chemotherapies and in monitoring cancer-related symptoms and infections. Therefore, further studies are warranted. Given its promising potential, piperine is likely to gain widespread acceptance for use in clinical trials targeting cancer, either as a standalone treatment or as a bioenhancer (93).

## 7 Conclusion

Piperine, a bioactive alkaloid derived from *Piper nigrum*, has demonstrated significant potential as a natural compound for the prophylactic and therapeutic management of cancer. Its multifaceted mechanisms of action target several hallmarks of cancer, including the suppression of proliferative signaling, induction of apoptosis, inhibition of angiogenesis and metastasis, modulation of autophagy, disruption of redox homeostasis, and regulation of immune responses. Additionally, piperine has shown a distinct ability to enhance drug bioavailability and overcome chemoresistance, making it a promising adjuvant in conventional chemotherapy. Furthermore, groundbreaking drug delivery systems, such as nanoparticles, micelles, and liposomes, have significantly enhanced the pharmacokinetics and therapeutic efficacy of these agents.

Despite mounting evidence of its pharmacological and anticancer properties, piperine still faces several limitations. Clinical evidence remains scarce, with most research confined to preclinical models and *in vitro* studies. Additionally, its low oral bioavailability and poor aqueous solubility may hinder its therapeutic application. Significant gaps remain in our understanding of piperine’s precise *in vivo* mechanisms, including its effects on the tumor microenvironment, redox balance, and immune-mediated cell death. Additionally, long-term safety studies, pharmacokinetic characterization, and standardized dosing protocols are lacking.

To determine piperine’s safety and potential as a cancer treatment or adjuvant agent, future research should focus on enhancing its bioavailability through advanced delivery methods, validating its efficacy *in vivo*, and conducting well-designed clinical trials.

In conclusion, piperine holds considerable promise as a multi-targeted, low-toxicity anticancer agent, with the potential to complement existing cancer treatments and make a meaningful contribution to integrative oncology.
